# Surface Coupling between Mechanical and Electric Fields Empowering Ni‐Rich Cathodes with Superior Cyclabilities for Lithium‐Ion Batteries

**DOI:** 10.1002/advs.202200622

**Published:** 2022-04-27

**Authors:** Zhongsheng Dai, Jianhang Wang, Huiling Zhao, Ying Bai

**Affiliations:** ^1^ International Joint Research Laboratory of New Energy Materials and Devices of Henan Province School of Physics and Electronics Henan University Kaifeng 475004 P. R. China; ^2^ Academy for Advanced Interdisciplinary Studies Henan University Kaifeng 475004 P. R. China

**Keywords:** electrochemical performance, finite element analysis, piezoelectric effect, polarization, stress–strain

## Abstract

Ni‐rich cathodes with high energy densities are considered as promising candidates for advanced lithium‐ion batteries, whereas their commercial application is in dilemma due to dramatic capacity decay and poor structure stability stemmed from interfacial instability, structural degradation, and stress–strain accumulation, as well as intergranular cracks. Herein, a piezoelectric LiTaO_3_ (LTO) layer is facilely deposited onto Li[Ni*_x_* Co_y_Mn_1−_ *_x_*_−_ *_y_*]O_2_ (*x* = 0.6, 0.8) cathodes to induce surface polarized electric fields via the intrinsic stress–strain of Ni‐rich active materials, thus modulating interfacial Li^+^ kinetics upon cycling. Various characterizations indicate that the electrochemical performances of LTO‐modified cathodes are obviously enhanced even under large current density and elevated temperature. Intensive explorations from in situ X‐ray diffraction technique, finite element analysis, and first‐principle calculation manifest that the improvement mechanism of LTO decoration can be attributed to the enhanced structural stability of bulk material, suppressed stress accumulation, and regulated ion transportation. These findings provide deep insight into surface coupling strategy between mechanical and electric fields to regulate the interfacial Li^+^ kinetics behavior and enhance structure stability for Ni‐rich cathodes, which will also arouse great interest from scientists and engineers in multifunctional surface engineering for electrochemical systems.

## Graphical abstract

This work provides deep insight into the surface coupling strategy between mechanical and electric fields to regulate the interfacial Li^+^ kinetics behavior and enhance structure stability for Ni‐rich cathodes by encapsulating Li[Ni*_x_* Co*_y_* Mn_1−_ *_x_*_−_ *_y_*]O_2_ secondary particles with piezoelectric LiTaO_3_ layer to improve their electrochemical performances by a facile sol–gel method.

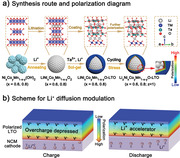

## 1 Introduction

The increasingly aggressive energy crisis and environmental issues have advanced the exploration and development of state‐of‐art lithium‐ion batteries (LIBs).^[^ ^1^, ^2^, ^3^ ^]^ Specifically, Ni‐rich Li[Ni*_x_* Co*_y_* Mn_1−_ *_x_*_−_ *_y_*]O_2_ (*x* ≥ 0.6) cathodes have been widely recognized as promising candidates for advanced LIBs due to their superior specific capacity (≈278 mAh g^−1^), high operating voltage (≈3.8 V), fast Li^+^ diffusion (≈10^−10^ cm^2^ s^−1^), and relatively low cost.^[^ ^4^, ^5^, ^6^ ^]^ Profiting from the marginal overlap of low‐spin Ni^2+/3+/4+^ (*e*_g_ band) with the top of O^2−^ (2*p* band), Ni^2+^ could be oxidized to Ni^4+^ without loss of lattice oxygen, and a discharge capacity well above 200 mAh g^−1^ might be achieved.^[^ ^7^ ^]^ Furthermore, low‐Co or Co‐free Ni‐rich cathodes could also alleviate political and ethical issues.^[^ ^8^ ^]^ However, with the lowest unoccupied molecular orbital, high‐valent transition metal (TM) ions (Ni^4+^ in particular) in a highly delithiated state tend to involve in parasitic reactions with lattice oxygen and electrolyte, which inevitably results in TM ions dissolution, lattice oxygen escape, irreversible phase transition, serious structural degradation as well as Li^+^/Ni^2+^ cation mixing.^[^ ^9^, ^10^ ^]^ From another perspective, structural degradation induced by drastic volume variation upon Li^+^ extraction/insertion leads to severe stress–strain accumulation.^[^ ^11^ ^]^ Then numerous microcracks will be generated and propagated along the grain boundaries of secondary particles, supplying microchannel for the liquid electrolyte to penetrate and induce more parasitic side reactions between cathode and electrolyte, finally leading to severe pulverization of secondary particles and rapid capacity decay of Ni‐rich cathodes.^[^ ^12^, ^13^, ^14^ ^]^ The higher the Ni content is, the more serious degradation of microcracks will be, especially when the Ni content exceeds 80%.^[^ ^15^ ^]^

To enhance the structural stability and electrochemical performances of Ni‐rich cathodes, various strategies including in situ/ex situ coating,^[^ ^16^, ^17^ ^]^ heteroatom doping,^[^ ^18^ ^]^ microstructure design,^[^ ^19^ ^]^ grain boundary engineering,^[^ ^20^ ^]^ and single‐crystal synthesis^[^ ^21^ ^]^ have been widely investigated, wherein surface coating is regarded as an effective approach due to their multi‐functions in protecting active material from direct contact with the electrolyte and suppressing the generation of microcracks.^[^ ^15^ ^]^ However, the coating layers are generally found apt to fall away from bulk Ni‐rich material under repeated cycling due to weak interaction, lattice mismatch, and dramatic anisotropic lattice contraction along the crystallographic *c*‐axis.^[^ ^22^ ^]^ Additionally, diffusion energy barriers at solid–solid (coating layer‐bulk cathode) and solid–liquid (coating layer‐liquid electrolyte) interfaces will impede Li^+^ diffusion and accumulate interfacial stress–strain, which also induces continuous microfracture aggravation.^[^ ^23^ ^]^ Consequently, multi‐functional surface coating approaches with the aim of depressing interfacial side reactions and alleviating strain accumulation to suppress bulk mechanical degradation and enhance the long‐term cycling performance of Ni‐rich cathodes have attracted extensive attention in the current research.^[^ ^24^, ^25^, ^26^, ^27^, ^28^ ^]^ Besides fast ion conductors and other lanthanum/titanium‐based oxides, several electro‐optical compounds with piezoelectric and ferroelectric properties have been implemented as modification materials for high‐performance batteries. For example, Huo et al. introduced a typical ferroelectric material of BaTiO_3_ to address the non‐uniform ion deposition in zinc (Zn) metal batteries.^[^ ^25^ ^]^ During electrochemical cycling, the piezoelectric BaTiO_3_ coating layer could cooperate with the stress–strain effect of Zn‐anode, and thus, the induced polarized electric field will benefit from the uniform deposition of Zn^2+^ ions. In our previous studies, it has been proved that the piezoelectric coating layer could boost the interfacial Li^+^ transportation in Li‐rich cathode upon electrochemical cycling.^[^ ^29^ ^]^

In view of the polarized electric field generated by piezoelectric material and the dramatic volumetric change of Ni‐rich cathodes upon cycling, typical piezoelectric material, LiTaO_3_ (LTO), with a piezoelectric coefficient of 14.6 pC N^−1^ and ionic conductivity of 10^−6^–10^−5^ cm^2^ s^−1^, was utilized to decorate the Ni‐rich materials. The modification mechanism of piezoelectric LTO coating onto Ni‐rich cathodes of Li[Ni_x_Co_y_Mn_1−x−y_]O_2_ (*x* = 0.6, *y* = 0.2, NCM622; *x* = 0.8, *y* = 0.1, NCM811) was systematically investigated (with similar morphology and particle size for comparison) to clearly establish the effect of stress–strain transformation in LTO on the electrochemical and structural stabilities of the bulk cathode. According to the crystallographic structures shown in Figure S1a,b, Supporting Information, the lattice mismatch of Ni‐rich cathode and LTO compound along the *c*‐axis was calculated to be only 2.8%, indicating superior compatibility between the bulk and external layer. In this case, the deposited LTO layer was expected to primarily act as a separator, protecting Ni‐rich particles from electrolyte scavenging and thus contributing to interfacial as well as electrochemical stabilities. As the main feature of this work, the intrinsic piezoelectric characteristic of the LTO coating layer was intensively concerned from the perspective of polarized electric field induced by the volume variation of Ni‐rich cathode and its influence on Li^+^ diffusion at the cathode–electrolyte interface during charge/discharge processes. The crystalline structure, surface chemical environment, and electrochemical performances of the pristine and LTO‐coated Ni‐rich cathodes were intensively investigated to explore the influence of LTO on NCM622 and NCM811. In situ X‐ray diffraction (XRD) analysis, finite element simulation, and first‐principle calculation further revealed the specific regulation effect of the polarized electric field from the piezoelectric LTO. This work not only establishes the multi‐functional layer of LTO in initiatively regulating ion transportation and contributing to electrochemical properties but also broadens the study and application interest in multi‐field coupling in energy storage and conversion systems.

## 2 Results and Discussion

**Figure 1:**
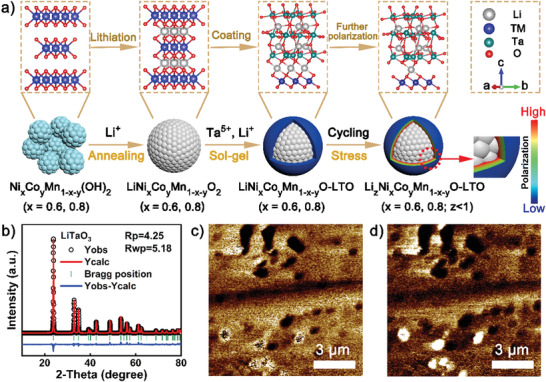
a) Schematic diagram of the preparation of LTO‐coated Ni‐rich cathode and the volumetric change influence of Ni‐rich material on the LTO coating layer; b) XRD pattern with Rietveld refinement data for the as‐prepared pure LTO; c) magnitude and d) phase images detected by PFM technique based on the piezoelectric response of the as‐synthesized LTO particles.

The preparation process of pristine and LTO‐modified Ni‐rich cathodes is schemed in **Figure** **1**a, in which the modification mechanism was proposed from the synergistic effect based on the physical separation and the piezoelectric polarization of the LTO coating layer. To confirm the piezoelectric property of the coating layer, pure LTO powder was synthesized using the same synthesis conditions as in the coating operation. XRD pattern with Rietveld refinement result (Figure 1b) displayed the ferroelectric phase of the as‐prepared LTO material without any other impurity. Scanning electron microscope (SEM) characterization (Figure S1c, Supporting Information) demonstrated that the particles of pure LTO powder were homogeneously distributed, with a diameter of ≈400 nm. A piezoelectric force microscope (PFM) with double alternating resonance tracking mode was employed to further investigate the piezoelectric property of the as‐prepared LTO material, with its working mechanism graphed in Figure S1e, Supporting Information, based on the inverse piezoelectric effect. Morphological image (Figure S1f, Supporting Information) probed by the PFM technique confirmed the particle size of the as‐prepared LTO; meanwhile, the high‐contrast amplitude and phase images manifested its desirable piezoelectric effect (Figure 1c,d).^[^ ^26^ ^]^

**Figure 2:**
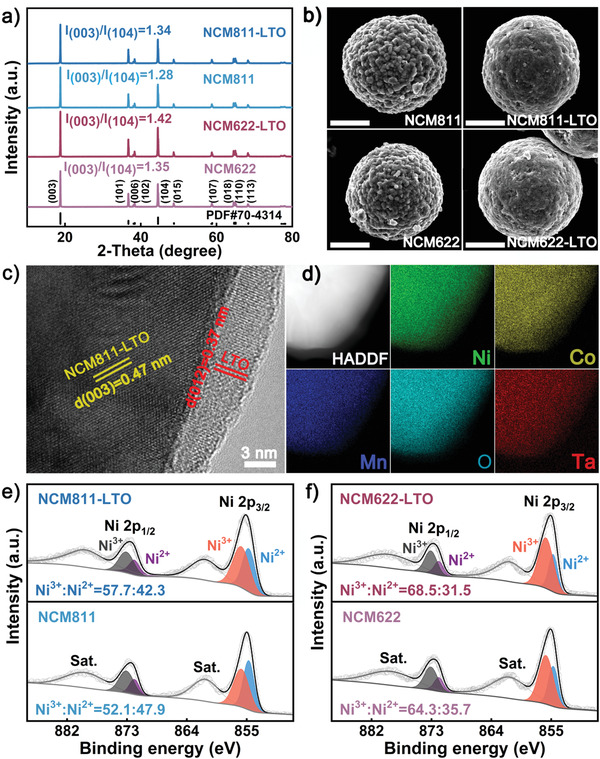
a) XRD patterns and b) SEM images of the as‐prepared NCM622, NCM622‐LTO, NCM811, and NCM811‐LTO with a scale bar of 5 µm; c) HRTEM and d) element mapping images of NCM811‐LTO; Ni 2p spectra of e) NCM811/NCM811‐LTO and f) NCM622/NCM622‐LTO.

To investigate the influence of the LTO coating layer on the crystallographic structure of bulk Ni‐rich cathode, powder XRD patterns of all the as‐synthesized samples were collected and shown in **Figure** **2**a. All diffraction peaks could be clearly indexed to hexagonal layered *α*‐NaFeO_2_ structure with a space group of *R‐3m* (JCPDS No. 70–4314), indicating that the LTO coating layer did not influence the crystal structure of bulk Ni‐rich cathodes,^[^ ^24^ ^]^ wherein the sharp diffraction peaks implied the excellent crystallization in all as‐prepared samples. In addition, the distinct splitting of (006)/(102) and (018)/(110) double peaks indicated the highly‐ordered layered structure for all samples. Generally, the intensity ratio of *I*_(003)_/*I*_(104)_ was admitted to present the Li^+^/Ni^2+^ cation mixing degree in Ni‐rich cathode,^[30,^ ^31^ ^]^ which were calculated to be 1.35, 1.42, 1.28, and 1.34 for NCM622, NCM622‐LTO, NCM811, and NCM811‐LTO respectively, indicating the alleviation of Li^+^/Ni^2+^ cation mixing in the LTO‐coated materials. This result was further confirmed by the Rietveld refinement analysis (Figure S2, Supporting Information), wherein the Li^+^/Ni^2+^ cation mixing degrees were determined to be 3.72%, 3.55%, 4.21%, and 3.95% for the corresponding materials. The lowered Li^+^/Ni^2+^ cation mixing degree by LTO modification will contribute to Li^+^ diffusivity and the resultant electrode performances, which will be discussed in the following context. As compared in Table S1, Supporting Information, lattice parameters *a* and *c* experienced little change, quantitatively indicating the intrinsically stabilized crystalline structure after LTO modification. To reveal the successful LTO deposition, magnified content of 10 wt.% LTO was conducted with the same procedure, and the corresponding XRD pattern is shown in Figure S1d, Supporting Information. Besides the characteristic peaks of Ni‐rich cathodes, the main diffraction lines of LTO clearly appeared (labeled by asterisks). Additionally, it could be easily observed that the particle surfaces of NCM622‐LTO and NCM811‐LTO (Figure 2b) became ambiguous compared with those of the pristine particles, indicating that the LTO layers were successfully coated on the surface of bulk Ni‐rich materials. Typically, in the high‐resolution transmission electron microscope (HRTEM) image of NCM811‐LTO (Figure 2c), the lattice fringes with an average space of ≈0.47 nm could be identified in the (003) plane of the layered structure, and the heterogeneous lattice fringes with a distance of ≈0.37 nm were ascribed to (012) plane of LTO.^[^ ^22^ ^]^ Moreover, the intimate contact between the inner (003) lattice fringe and that of the external (012) clearly confirmed the desirable lattice matching, as well as interface compatibility. The energy dispersive spectra (EDS) mappings of Ni, Mn, Co, O, and Ta elements were meanwhile collected and shown in Figure 2d, from another perspective illustrated the successful coating of piezoelectric LTO layer onto Ni‐rich secondary particles. Especially, the cross‐section morphology of NCM811‐LTO and the corresponding EDS mapping images (Figure S3, Supporting Information) clearly revealed that the Ta element was homogeneously distributed, providing direct evidence for the homogeneous LTO surface modification realized.

To detect the elemental composition and chemical environment of the as‐prepared cathodes, X‐ray photoelectron spectroscopy (XPS) characterization was conducted, and the results were calibrated with C 1s of 284.4 eV (Figure 2e,f and Figure S4, Supporting Information). As shown in the surveys, the signals of the Ta element appeared, which could be attributed to Ta 4f_5/2_ and Ta 4f_7/2_ spin‐orbit doublet with binding energies of 27.5 and 25.6 eV in the pentavalent Ta^5+^ oxidation state, respectively (Figure S4a,b, Supporting Information).^[^ ^32^ ^]^ The signals of Ni 2p could be divided into four peaks at 854.8 and 872.1 eV, 856.3 and 875.3 eV, which were ascribed to 2p_3/2_ and 2p_1/2_ of Ni^2+^ and Ni^3+^ cations, respectively, indicating the coexistence of Ni^2+^ and Ni^3+^ ions in all the as‐prepared materials.^[^ ^27^ ^]^ Further analysis suggested that the ratios of Ni^3+^/Ni^2+^ in NCM811‐LTO (Figure 2e) and NCM622‐LTO (Figure 2f) were both larger than those in the pristine samples, reflecting more Ni^2+^ ions oxidized by oxygen vacancies under high‐temperature annealing in the LTO coating procedure (Figure S4c,e, Supporting Information). The increased Ni^3+^/Ni^2+^ ratios on the surface of modified Ni‐rich materials could effectively suppress the formation of electrochemically inactive rock‐salt phase by reducing Li^+^/Ni^2+^ mixing,^[^ ^16^ ^]^ thus contributing to electrochemical performance improvement. Moreover, the ratios of Li_2_CO_3_ and lattice O1s spectra, located at 532.2 and 529.1 eV, were obviously decreased after LTO modification (Figure S4c,e, Supporting Information), implying the surface by‐products were favorably suppressed.^[^ ^32^ ^]^ Simultaneously, the decreased alkaline content in the surface of NCM811‐LTO and NCM622‐LTO was further verified from the analysis of Li_2_CO_3_ (Figure S4d,f, Supporting Information), which was believed to be originated from the neutralization reaction of alkaline residual lithium compounds with acidic substances in LTO coating process, and could effectively weaken the gas production upon electrochemical cycling.^[^ ^33^ ^]^

**Figure 3:**
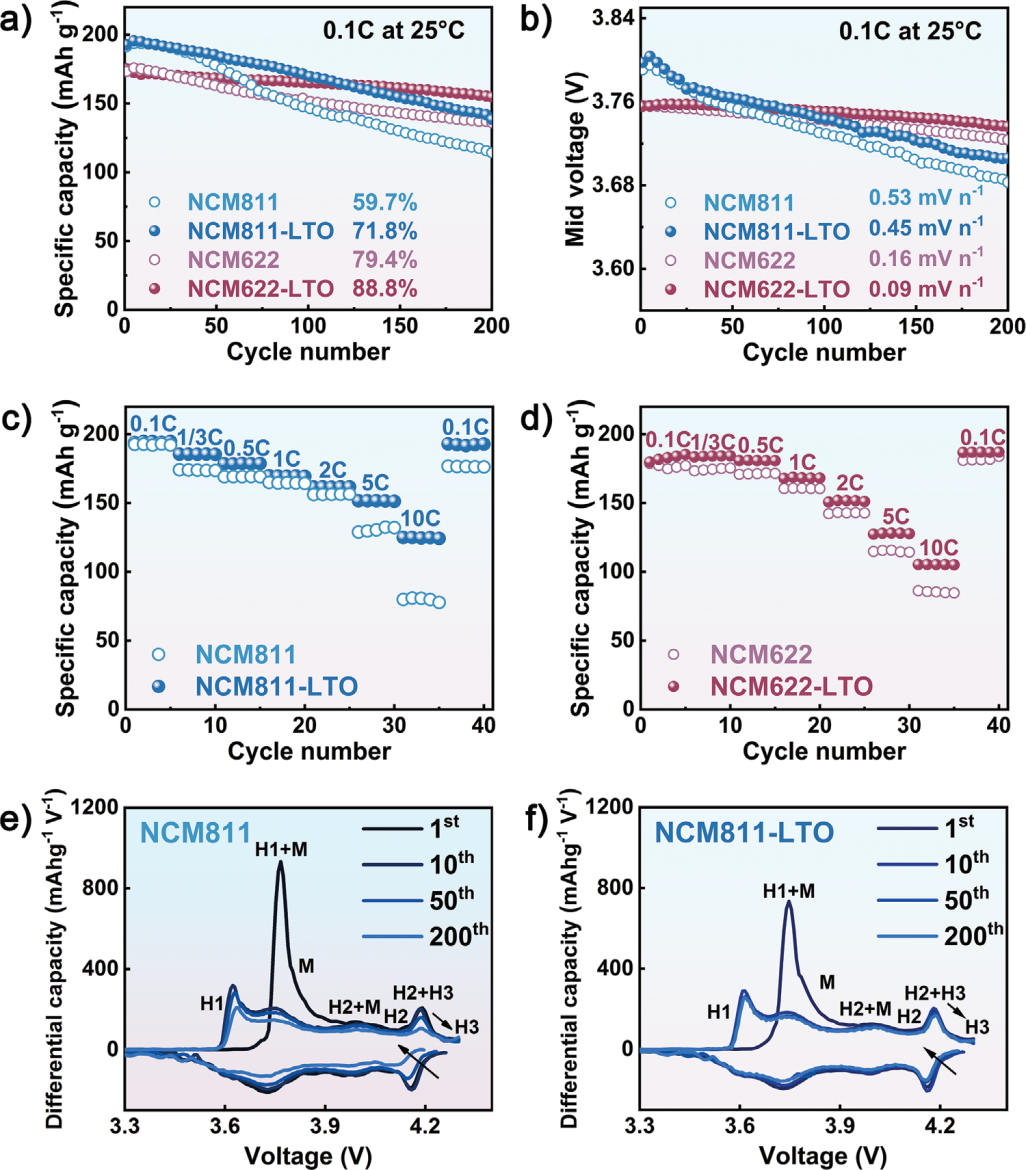
Cycling performances at 0.1 C and 25 ° C a) and voltage fading curves b) measured for the as‐prepared cathodes; rate performances for c) NCM811/NCM811‐LTO and d) NCM622/NCM622‐LTO; *dQ/dV* curves for e) NCM811 and f) NCM811‐LTO at 2.7–4.3 V.

To demonstrate the influences of piezoelectric LTO coating on the electrochemical performances of Ni‐rich materials, the electrodes were investigated at the voltage range of 2.7–4.3 V. As can be observed in **Figure** **3**, Figure S5 and Table S2, Supporting Information, the electrochemical properties of the modified cathodes were obviously improved after LTO surface modification, which was more prominent for those cathodes with higher Ni content and under higher temperature operation. In these cases, a larger extent of volume change will be induced from NCM cathodes, with stronger stress–strain resonance in LTO, contributing more extra electric field force through mechanic–electric coupling to influence the Li^+^ transportation.

Careful investigation of the initial charge–discharge curves and d*Q*/d*V* profiles between the cathodes before and after LTO decoration indicated that the capacities were originated from similar redoxes (Figure 3e,f and Figure S5a,b,e,f, Supporting Information). Particularly, the reversible phase transition of hexagon 2 (H2) to hexagon 3 (H3) could be found for NCM811 electrodes (Figure 3e,f), which was absent in NCM622 materials (Figure S5e,f, Supporting Information), indicating more active Ni ions could be participated in electrochemical reaction for higher Ni content cathodes. This phase transition was believed to trigger the dramatic anisotropic contract along the *c*‐axis, which induced serious mechanical stress–strain leading to the generation of microcracks on particles and eventually resulting in irreversible structural degradation and rapid capacity decay.^[^ ^16^, ^17^ ^]^ For the pristine NCM811 electrode, the intensity of H2—H3 redox rapidly declined with the polarization (the potential gap between cathodic and anodic peaks) significantly increased, implying the irreversible structural degeneration upon cycling (Figure 3e).^[^ ^19^ ^]^ In comparison, the NCM811‐LTO electrode exhibited remarkably‐enhanced reversibility in the H2—H3 phase transition (Figure 3f), which was consistent with the improved cycling stability in Table S2, Supporting Information. Similarly, the alleviated irreversibility of phase transition and polarization could be obtained for the NCM622‐LTO cathode, again manifesting the contribution of LTO in the structural stability of Ni‐rich cathodes.

To investigate the influence of LTO modification on the kinetics behaviors of NCM materials, electrochemical impedance spectroscopies (EIS) of all the as‐prepared cathodes were collected, and the corresponding Nyquist plots are shown in Figure S6, Supporting Information. Each profile was composed of two semicircles and one slope line, wherein the first semicircle in the high‐frequency region corresponded to the surface‐film resistance (*R*_sf_) associated with Li^+^ transportation through cathode electrode interface (CEI), the second semicircle in the intermediate frequency region was assigned to the charge transfer resistance between CEI and active material (*R*_ct_), and the sloped line in the low‐frequency region could be ascribed to the Warburg impedance connected with Li^+^ diffusion in the bulk cathode. In addition, the high‐frequency intercept of the Nyquist plot with the real axis (*Z*′) represented the solution resistance (*R*_s_).^[^ ^33^ ^,34]^ The equivalent circuit is schemed in the inset of Figure S6c, Supporting Information, and the fitting values of *R*_sf_ and *R*_ct_ are listed in Table S3, Supporting Information, as well as the corresponding normalized resistances are compared in Figure S6c,d, Supporting Information. It should be noted that the growth of *R*_sf_ and *R*_ct_ values with cycling were effectively suppressed after LTO modification. On one hand, the deposited LTO layer will shield active material from corrosion by acid electrolyte and thus alleviate possible side reactions, which will be further confirmed in the lattice microstructure observation after cycling. From another perspective, the intrinsic piezoelectric resonance of LTO might generate an additional field to afford the attached driving force for Li^+^ diffusion at the cathode–electrolyte interface.

Cyclic voltammetry (CV) tests at different scan rates were also carried out and the obtained spectra for all cathodes are demonstrated in Figure S7a,d, Supporting Information. Typically, each electrode exhibited larger polarization under higher scan rates, which were found lowered for LTO modified cathodes compared with their pristine counterparts. According to the relationship between scan rates (*v* ^1/2^) and peak current (*i*_p_) (Figure S7e,f, Supporting Information), the Li^+^ diffusion rates ($D_{Li^{+}}$) could be calculated using the Randles–Sevcik equation:

(1) $$i_{p}=\;2.69\;\times 10^{5}n^{3/2}AC_{0}{D_{Li^{+}}}^{1/2}\nu^{1/2}$$

where *n* is the electron number in the redox reaction (*n* = 1), *A* is the geometric area of the cathode assembled in the cell (0.5024 cm^2^), *C*_0_ is the initial molar concentration of Li^+^ ions (5.26 × 10^−3^ mol cm^−3^), $D_{Li^{+}}$ is the Li^+^ diffusion coefficient, and *v* is the scan rate.^[^ ^35^ ^]^ Thus obtained $D_{Li^{+}}$ are compared in Figure S6e,f and Table S4, Supporting Information. It could be found that both modified cathodes exhibited higher Li^+^ diffusion rates than their pristine electrodes under the electrochemical process, coinciding with the previous EIS analysis. The $D_{Li^{+}}$ analysis herein not only explained the enhanced rate performance in Figure 3 but also confirmed the greater contribution to kinetics and electrochemical performances of higher Ni content cathode by surface LTO layer.

**Figure 4:**
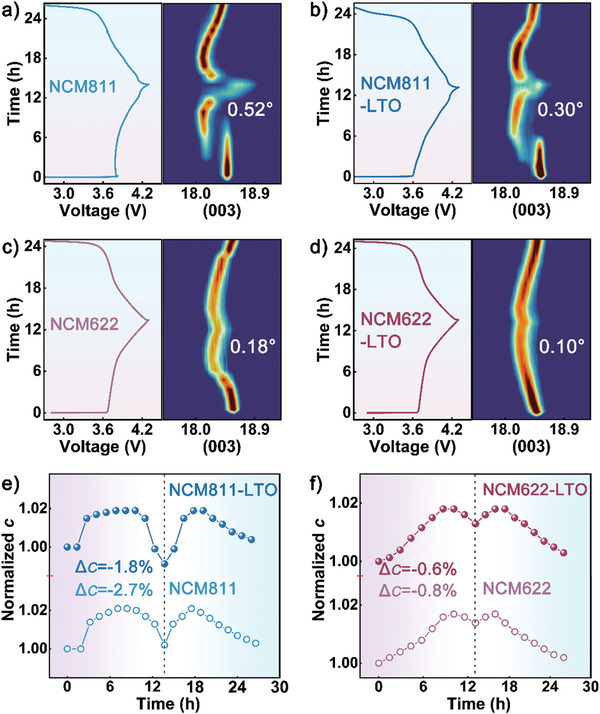
In situ XRD patterns of the (003) diffraction peaks in the initial cycle for a) NCM811, b) NCM811‐LTO, c) NCM622, and d) NCM622‐LTO; normalized evolutions of the lattice parameter *c*: e) NCM811/NCM811‐LTO and f) NCM622/NCM622‐LTO.

To reveal the influence of the piezoelectric LTO coating layer on the crystallographic structure evolution of Ni‐rich cathodes, in situ XRD characterizations were carried out in the voltage range of 2.7–4.3 V under 0.1 C. **Figure** **4** and Figure S8, Supporting Information, exhibits the contour plots for the (003) and (101) diffraction peaks for all as‐prepared cathodes, in which the normalized lattice changes along the *c‐* and *a‐*axis are also presented. As can be observed in Figure 4a–d, the (003) peaks unexceptionally shifted to lowered diffraction angle under 4.18 V, followed by reversely moving to higher 2*θ* when further charged to 4.3 V. As calculated and compared in Figure 4e,f, the lattice parameter *c* underwent a nonlinear evolution composed of gradually increasing and continuously degradation, with the voltage 4.18 V as a transition point. The *c*‐axis expansion under 4.18 V could be attributed to the increase of O—O repulsion due to Li^+^ removal within the Li‐slab^[^ ^36^, ^37^ ^]^ and the drastic lattice shrink above 4.18 V could be explained by the negative charge transfer from O to Ni ions at high state‐of‐charge, reducing the columbic repulsive force in O—Li—O slab along *c*‐axis and crystalline structure degradation during H2—H3 phase transition for NCM811 material.^[^ ^24^ ^]^ The (003) peak shifts were determined to be 0.52° and 0.30° during the H2—H3 phase transition for the NCM811 and NCM811‐LTO cathodes, with the abrupt parameter *c* shrink of 2.7% and 1.8%, respectively. As for the NCM622 cathodes, the shift of the (003) peak decayed from 0.18° to 0.10° after LTO decoration, corresponding with parameter *c* declined from 0.8% and 0.6%, respectively. The contribution of LTO modification to lattice stabilization revealed herein could be attributed to the specific piezoelectric effect, which will be discussed in the following mechanism analysis. It should be emphasized that the deposited LTO contributed more in stability for higher Ni content material, from another viewpoint reflecting the more effectiveness in depressing lattice oxygen escapes at the surface of NCM811. Figure S8a,d, Supporting Information shows that the (101) diffraction lines for all electrodes are monotonously shifted to a higher angle in the charging process, indicating a gradual decrease in the lattice parameter *a* (Figure S8e,f, Supporting Information), which could be attributed to the oxidation of Ni^2+^→Ni^4+^.^[^ ^38^ ^]^ It is worth noting that larger shrinkage of the *a*‐axis was found for the cathode with higher Ni content (Figure S8e,f, Supporting Information), further manifesting that more Ni ions were involved in the electrochemical reaction.

**Figure 5:**
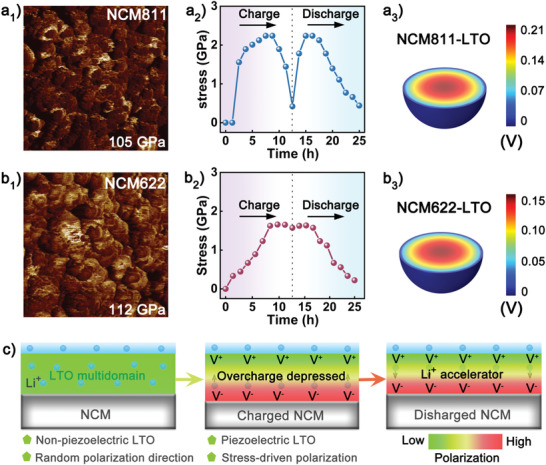
Young's modulus of the pristine samples detected by AFM technique: a_1_) NCM811 and b_1_) NCM622; stress curves of the LTO‐modified samples in the initial cycle: a_2_) NCM811‐LTO and b_2_) NCM622‐LTO; finite element analysis results of polarized electric field distribution from LTO layer under the compression strength with the corresponding stress of Ni‐rich cathode at fully discharged state: a_3_) NCM811‐LTO and b_3_) NCM622‐LTO; c) schematic diagrams for the polarization of LTO and its regulating effect on Li^+^ diffusion upon cycling.

Atomic force microscopy (AFM) was utilized to characterize Young's moduli of NCM811 and NCM622, which were determined to be 105 and 112 GPa (**Figure** **5**a_1_,b_1_), respectively, with the corresponding morphological images provided in Figure S9, Supporting Information. Based on the varied lattice parameter *c* analyzed by in situ XRD, the stress and strain values of NCM622‐LTO and NCM811‐LTO cathodes in the initial cycle were further calculated using formulas (S1) and (S2), Supporting Information, which are further compared in Figure 5a_2_,b_2_ and Figure S10a,b, Supporting Information, respectively. All curves increased in the voltage range of 2.7–4.18 V and then degraded until the cut‐off voltage of 4.3 V, which declined more dramatically for the NCM811‐LTO cathode. This should be attributed to the H2—H3 phase transition experienced by high Ni content particles as indicated in d*Q*/d*V* profiles (Figure 4e,f), suffering more severe structural instability upon long‐term cycling. With the transportation of expansion and contraction of interior Ni‐rich particles, the induced force in piezoelectric LTO will initiatively motivate the additional polarized electric field, which can be stimulated and shown in Figure 5a_3_,b_3_ and Figure S10c,d, Supporting Information. In the charged state, the maximum values of piezoelectric potential triggered in NCM622‐LTO and NCM811‐LTO cathodes were determined to be 0.40 and 0.51 V, respectively, with the orientation opposite to the Li^+^ diffusion. The same maximum potential could be obtained for the discharge process, with the extra voltage of 0.15 and 0.21 V for NCM622 and NCM811 maintained at the end of the first cycle, respectively. It should be emphasized that the orientation of triggered potential was in alignment with that of Li^+^ diffusion in the discharge process, which is favorable for Li^+^ kinetics behavior in battery operation.

Based on the above analysis, the schematic diagrams of LTO contribution to surface kinetics are demonstrated in Figure 5c, clearly illuminating the regulation effect of the piezoelectric LTO layer on the Li^+^ diffusion at the cathode–electrolyte interface. As an intrinsically multi‐domain crystal, the surface deposited LTO material fails in exhibiting specific piezoelectric properties due to the randomly distributed orientations for individual single domains.^[^ ^39^, ^40^ ^]^ Nevertheless, piezoelectric characteristics could be triggered in the LTO layer when it is subjected to the severe strain of internal NCM811. Particularly, during the charge process, the overwhelming external voltage (2.7–4.3 V) dominated the Li^+^ transportation compared with the additional polarized electric field, whereas the polarized electric field might regulate the fast extraction of Li^+^ to alleviate lattice degradation at high state‐of‐charge (Figure S11, Supporting Information) and contribute to anti‐overcharge when the battery was abused somehow. During the discharge process, the polarized potential was of the same orientation as that of the external electric field, which acted as an accelerator to promote Li^+^ diffusion at the electrolyte–cathode interface. Simultaneously, the improved Li^+^ kinetics could depress the stress–strain accumulation by lowering the interfacial energy barrier and eventually elevating the electrochemical performances of the Ni‐rich cathodes. It should be noted that the LTO could not be intrinsically polarized even under an applied voltage of 5 V (Figure S12, Supporting Information), thus its polarization induced by the external electric field (2.7–4.3 V) could be ignored.

**Figure 6:**
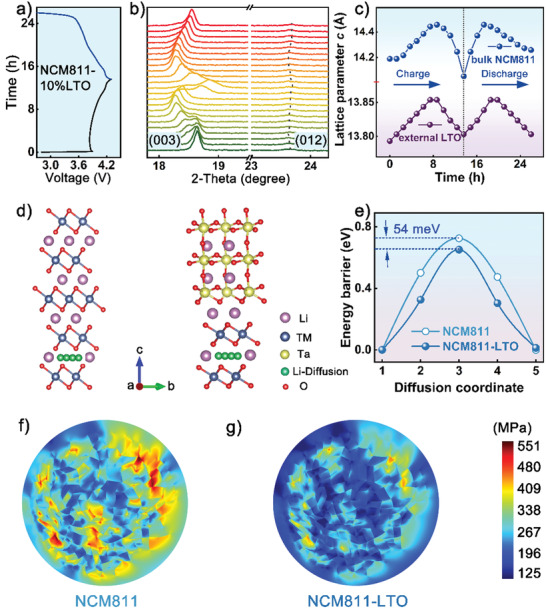
Charge/discharge curves a) and in situ XRD patterns b) of the LTO‐modified NCM811 cathode with magnified coating content of 10 wt.% in the initial cycle; c) the variation of the lattice parameter *c* for NCM811‐10 wt.% LTO upon cycling; d) theoretical models for the Li^+^ diffusion paths and e) corresponding diffusion energy barriers for NCM811 and NCM811‐LTO; f) tension stress distribution for NCM811 and g) NCM811‐LTO particles at the maximum strain state.

To clarify that the polarization electric field of the external LTO coating layer could be triggered by the internal cathode, in situ XRD test (**Figure** **6**a) was performed on the modified Ni‐rich material with higher Ni content (NCM811), and a larger LTO coating proportion (magnified to 10%). Due to the shielding effect of the aluminum window, only the (012) diffraction peak of LTO can be observed in Figure 6b, which was related to the (012) crystal plane comprising Ta atoms in the cell diagram (Figure S13, Supporting Information). The polarization of LTO was generally attributed to the deviation of Ta or Li atoms from the original position.^[^ ^41^ ^]^ Herein the prominent shift of the (012) line indicated the position variation of Ta atoms during charging and discharging processes, further confirming the generation of the polarized electric field in the LTO coating layer originated from the deviation of the Ta—O octahedral center. Careful observation in Figure 6b indicates that the peak shift of (012) for the LTO coating layer is accompanied by that of (003) for bulk NCM811, which is further exhibited in the normalized lattice parameter calculations in Figure 6c. It should be noted that though the (012) diffraction peak of the LTO layer fluctuated in integrated cycling, its corresponding lattice parameter was found always positively deviated from its original value. This result explained the continuous variation of the polarized electric field upon cycling, with its orientation remaining unchanged, coinciding with the previous simulation analysis in Figure 5a_3_,b_3_ and Figure S10c,d, Supporting Information. Herein, the excellent consistency in lattice evolution (Figure 6c) further verified the desirable compatibility between the piezoelectric LTO and bulk Ni‐rich layered material.

To establish the underlying influence of the LTO coating layer, density functional theory (DFT) calculation was applied to investigate the Li^+^ diffusion barrier for NCM811 and NCM811‐LTO cathodes, respectively. Based on the theoretical model of the Li^+^ migration pathway in the bulk Ni‐rich particle (Figure 6d), DFT calculation results indicated that a diffusion barrier drop of 54 meV could be obtained after LTO modification (Figure 6e), partly explaining the enhanced Li^+^ diffusion coefficient in CV analysis (Figure S7, Supporting Information) as well as the improved rate performances (Figure 3c,d). Finite element analysis was conducted to demonstrate the stress–strain distribution in NCM811 and NCM811‐LTO secondary particles under the maximum stress state, with their cross‐sections shown in Figure 6f,g. The NCM811 cathode showed large stress distributed in vast areas, which could be mainly attributed to the abrupt collapse of the *c*‐axis and the shrinkage of cell volume during the phase transition of H2—H3. Comparatively, the stress accumulation was obviously alleviated for NCM811‐LTO secondary particles, consistent with the previous in situ XRD analysis (Figure 4).

The enhanced structural stability after LTO surface decoration could be clearly illustrated by morphology characterizations after repeated cycling. As could be observed in the topographies of NCM811 and NCM811‐LTO cathodes after 200 cycles (Figure S14a, Supporting Information), the secondary particles of pristine NCM811 cathode experienced severally broken, exposing more primary grains into the electrolyte, which would continuously consume electrolyte and induce series parasitic side reactions. After the same electrochemical treatment, most of the NCM811‐LTO secondary particles maintained their integrity (Figure S14b, Supporting Information), providing solid evidence of structural stability enhancement, and the concerning suppression of lattice collapse as well as stress–strain accumulation.^[^ ^42^ ^]^ Additionally, HRTEM images were collected to compare the surface microstructure evolution of NCM811 and NCM811‐LTO cathodes after 200 cycles, which are carefully analyzed and compared in Figure S14c,d, Supporting Information. After repeated cycling, three distinct regions could be distinguished in the near‐surface of NCM811 (Figure S14c, Supporting Information). Besides the characteristic crystal texture of layered bulk structure in the interior (labeled as region I), two extra regions could be clearly observed. The medium region II, with a thickness of ≈5 nm, could be identified as NiO‐like compounds, which were believed to originate from the severe Li^+^/Ni^2+^ cation mixing and dissolution of transition metal ions.^[^ ^43^ ^]^ Additionally, an amorphous dense layer (≈3 nm) appeared at the outermost of the NCM811 particle, corresponding to the CEI component generated from surface side reactions. The electrochemically generated rock‐salt and CEI surface layers (regions II and III) have been reported to substantially inhibit Li^+^ transportation at the particle surface.^[^ ^44^ ^]^ As shown in Figure S14d, Supporting Information, the surface microstructure of NCM811‐LTO fairly remained compared with its uncycled counterpart in Figure 2. Clearly, the internal and external lattice fringes in regions I and II, with average spaces of ≈0.23 and ≈0.25 nm, could be unambiguously identified to (104) plane of Ni‐rich layered cathode (*R‐3m* space group) and (110) plane of LTO (*R‐3c* space group). The above HRTEM results confirmed that the LTO modification stabilized the surface structure and component, providing a favorable surface environment for ion transportation under electrochemical treatment.^[^ ^45^ ^]^ In addition, element mapping was applied to investigate the distribution of Ni, Co, Mn, O, and Ta elements on the surface of cycled NCM811‐LTO. As shown in Figure S14e, Supporting Information, the element distribution maintained homogeneous after 200 cycles, from another viewpoint confirming the strong compatibility between the bulk NCM811 and LTO coating layer. The above characterization results clearly established that the piezoelectric LTO coating could not only suppress the intergranular cracks and adverse evolution of structural degradation but also stabilize the near‐surface microstructure of Ni‐rich cathode to maintain its electrochemical performances.

## 3 Conclusion

In this work, the Ni‐rich secondary particles were successfully encapsulated by a piezoelectric LTO layer to improve their electrochemical performances through a facile sol–gel method. Typically, the NCM811‐LTO cathode exhibited a capacity retention of 71.8% after 200 cycles under 0.1 C, much higher than its pristine counterpart (59.7%). Additionally, the NCM811‐LTO delivered a higher discharge capacity of 129.9 mAh g^−1^ even under 10 C, in sharp contrast with the pristine cathode (79.8 mAh g^−1^). The enhanced electrochemical performances and rate capacities synchronously implied that the structural stability and Li^+^ kinetics of Ni‐rich cathodes might be improved by LTO decoration. In situ XRD, finite element analysis, and morphology characterizations after cycling elucidated that the bulk and surface structure stabilities could be effectively improved by alleviating the accumulation of stress–strain as well as the shielding effect of the LTO layer. The boosted kinetics behavior with the LTO layer could be attributed to the locally induced polarized electric field from the piezoelectric property of LTO and the lowered diffusion barrier by EIS investigation, CV detection, as well as first‐principles calculation. The positive effect of the introduced piezoelectric LTO surface layer on the Ni‐rich cathode could be summarized in the following aspects: 1) furnishing a local controller at the interface to regulate the Li^+^ diffusion during charge/discharge processes; 2) in situ converting and exploiting the inevitable stress–strain of bulk active material by the intrinsic piezoelectric characteristic of LTO decoration layer, thus contributing to crystal structure stability; 3) effectively alleviating lattice collapse under highly delithiated state in Ni‐rich materials through introducing local electric field; 4) serving as a physical separator to ameliorate the interfacial instability through avoiding any possible side reactions between electrode and electrolyte. The multi‐functions of piezoelectric LTO modification established herein not only provide an effective and pragmatic strategy for accelerating their commercial application through enhancing the structural and electrochemical stabilities but also paves the way to motivate the development of next‐generation electrode materials in taking full advantage of multi‐filed coupling approach, such as mechanical–electric filed coupling, based on the intrinsic physical characteristic of the active material as well as an artificial interface layer.

## Conflict of Interest

The authors declare no conflict of interest.

## Supporting information

Supporting Information

## Data Availability

The data that support the findings of this study are available from the corresponding author upon reasonable request.
