# Supplementary material for: Surface Coupling between Mechanical and Electric Fields Empowering Ni‐Rich Cathodes with Superior Cyclabilities for Lithium‐Ion Batteries
Source: Adv Sci (Weinh). 2022 Apr 27;9(18):2200622. doi: 10.1002/advs.202200622 (PMC9218785; doi:10.1002/advs.202200622)
Supplement: Supplementary file 1 — Supporting Information [file ADVS-9-2200622-s001.pdf]

Supporting Information

Surface Coupling between Mechanical and Electric Fields Empowering Ni-rich Cathodes with Superior Cyclabilities for Lithium-ion Batteries

Zhongsheng Dai,[1] Jianhang Wang,[1] Huiling Zhao,[1,2] Ying Bai[1,2]**

Z. Dai, J. Wang, Dr. H. Zhao, Prof. Y. Bai

[1]International Joint Research Laboratory of New Energy Materials and Devices of Henan Province, School of Physics and Electronics, Henan University, Kaifeng, 475004, P. R. China

[2]Academy for Advanced Interdisciplinary Studies, Henan University, Kaifeng, 475004, P. R. China

*Corresponding author. E-mail: zhao@henu.edu.cn (H. Zhao); ybai@henu.edu.cn (Y. Bai)

Experimental section

***Material synthesis*:** The commercial $[Ni_xCo_yMn_{1-x-y}](OH)_2$ (x = 0.6, 0.8) precursor and $LiOH{\cdot}H_2O$ (99.9%, Aladdin) powder were uniformly mixed with a molar ratio of 1:1.05, then annealed at 780 °C for 15 h under oxygen atmosphere with the heating rate of 2 °C min^{-1} to obtain the pristine $LiNi_{0.6}Co_{0.2}Mn_{0.2}O_2$ (NCM622) and $LiNi_{0.8}Co_{0.1}Mn_{0.1}O_2$ (NCM811). In the modification procedure, the different coating contents (1 wt.%, 2 wt.%, 3 wt.%, *etc.*) of $LiTaO_3$ (LTO) were applied and final optimize ratio of LTO *vs.* pristine NCMs was determined to be 2:100. Here, taking 2 wt.%-LTO coated NCM811 as an example, 0.0055 g CH_3COOLi (99.9%, Aladdin), 0.0374 g Ta_2O_5 (99.9%, Aladdin), 0.0127 g $C_4H_6O_6$ (99.9%, Aladdin) and 1.00 g NCM811 powders were dissolved into alcohol, respectively, then the above solution were dropped into NCM811 suspension, and the mixed solution was heated at 70 °C until the solvent was completely evaporated. The obtained samples was dried at 100 °C for 12 h, and annealed at 500 °C for 24 h with the heating rate of 1 °C min^{-1}, finally the sample of 2 wt.% coated NCM811 was obtained and named as NCM811-LTO. Therefore, NCM622-LTO and NCM811-LTO samples with LTO content of 2 wt.% were herein selected for intensive analysis and discussion in the following work.

***Physical characterizations*:** The crystal structures of all as-prepared materials were identified by X-ray diffraction (XRD, Bruker D8 Advance) with Cu-Kα radiation at a scan step of 0.02°, and the Rietveld refinement of XRD patterns was achieved by the Fullprof program. The morphologies of all as-prepared samples were observed by field-emission scanning electron microscope (FESEM, Geminisem 500) with the acceleration voltage of 10 kV. The detailed structural features and element mappings of all samples were obtained by high-resolution transmission electron microscope (TEM, FEI Talos F200X) with the acceleration voltage of 200 kV. The piezoelectric effect of as-prepared sample was measured by piezoelectric force microscopy (PFM, Oxford MFP3D). The Young's modulus of sample was determined by atomic force microscope (AFM, Bruker Senta, CA, USA). In a typical test, the as-synthesized powders were pressed into pellets, which were then fixed on a high-purity silicon wafer for AFM test with contact mode. The chemical environments of various elements consisted in cathode materials were characterized by X-ray photoelectron spectroscopy (XPS, Axis), further all XPS spectra were calibrated with the binding energy peak of C 1s posited at 284.8 eV.

***Electrochemical measurements*:** To be assembled as positive electrode in coin cell, the as-synthesized cathode material was mixed with super-P carbon black and polyvinylidene fluoride (PVDF) in a weight ratio of 8:1:1, and dissolved into N-Methyl-2-pyrrolidinone

(NMP). The obtained slurry was coated onto aluminum foil, roll-pressed, and dried at 90 °C in vacuum box. Then, the electrode was cut into circular pieces with the diameter of 8 mm and with mass loading of cathode active material near to 5.5 mg (~ 10.92 mg cm^{-2}) with the thickness of ~ 200 μm. The as-prepared electrode were assembled into CR2032-type coin in an Ar-filled glove box (MIKROUNA) for the subsequent electrochemical measurements, with Li metal as the reference electrode and Celgaed 2400 film as the separator. The electrolyte was prepared through dissolving 1 M $LiPF_6$ into ethylene carbonate (EC) and dimethyl carbonate (DMC) with volume ratio of 1:1. All galvanostatic charging/discharging tests were performed at different current densities in the same voltage ranges of 2.7–4.3 V (*vs.* Li^+/Li), using LAND battery test system (Wuhan, China) at room temperature and elevated temperature of 50 °C, respectively. In addition, rate performances were tested by a Neware battery testing system (Shenzhen, China) within a voltage range of 2.7–4.3 V (vs. Li^+/Li) at different current rates (1C = 220 mA g^{-1}). The electrochemical impedance spectra (EIS) of all samples were measured using an electrochemical workstation (CHI 600E, Shanghai, China) at the charged state of 4.3 V with an amplitude of 5 mV and in the frequency range from 10 mHz to 100 kHz. The cyclic voltammetry (CV) profiles were recorded at the scanning rate of 0.1 mV s^{-1} between 2.7 and 4.3 V on the electrochemical workstation (CHI 600E, Shanghai, China). Additionally, in situ XRD patterns of cathode electrodes were tested in the specially made chamber (Beijing scistar technology Co. Ltd) with the step of 0.013° in the scanning range of 10–55°.

***First principle calculations*:** First principle calculations were based on density functional theory (DFT) and performed within the Vienna ab initio simulation package (VASP).[1] For the optimization of crystal structure, Brillouin zone employed a 3 × 3 × 1 Gamma-center K-point grid for sampling and the cut off energy of plane wave is 520 eV. The convergence tolerances of energy and force were set as 10^{-5} eV and 0.02 eV $Å^{-1}$, respectively. The climbing image nudged elastic band (CI-NEB) method was applied to determine the diffusion energy barrier and the minimum energy pathway for Li^+ diffusion was explored in the materials, and the transition states were finally obtained by relaxing the force below 0.03 eV $Å^{-1}$.[2]

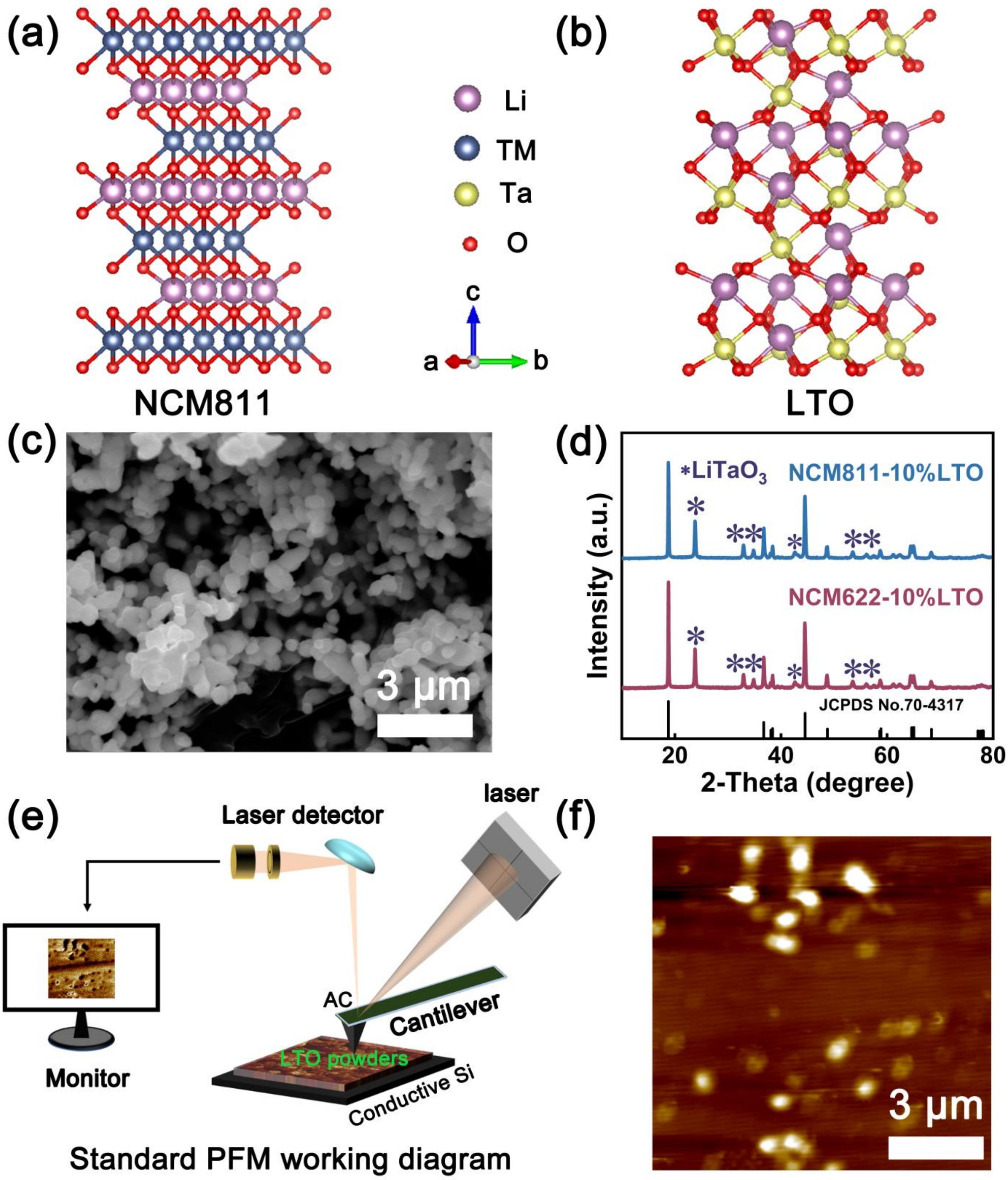

Figure S1. (a)(b) Crystal structures of the Ni-rich material and piezoelectric $LiTaO_3$ (LTO), respectively; (c) scanning electron microscope (SEM) image of the pure LTO material synthesized under same experimental conditions in the modification operation; (d) X-ray diffraction (XRD) patterns of the NCM811-10 wt.% LTO and NCM622-10 wt.% LTO samples; (e) schematic diagram for the work principle of piezoelectric force microscope (PFM); (f) morphological image of the pure LTO powder tested by PFM with the polarization voltage of 20 V.

On account of the similar lattice parameters of NCM811 and NCM622, NCM811 sample was taken as a representative to calculate the lattice matching degree between NCM811 and LTO along *c*-axis, using the following formula[3]:

$$\partial = \frac{c(\mathrm{NCM811}) - c(\mathrm{LTO})}{c(\mathrm{NCM811})} \qquad (1)$$

wherein *c* is the lattice parameter and ∂ represents the lattice mismatch degree. The *c* values were obtained by Rietveld refinement for the as-prepared NCM811 and LTO materials, which were determined to be 14.189 and 13.783, respectively. Thus the lattice mismatch (∂) of NCM811 and LTO was calculated to be 2.8%, indicating the excellent interface compatibility between piezoelectric LTO coating layer and bulk Ni-rich cathode.

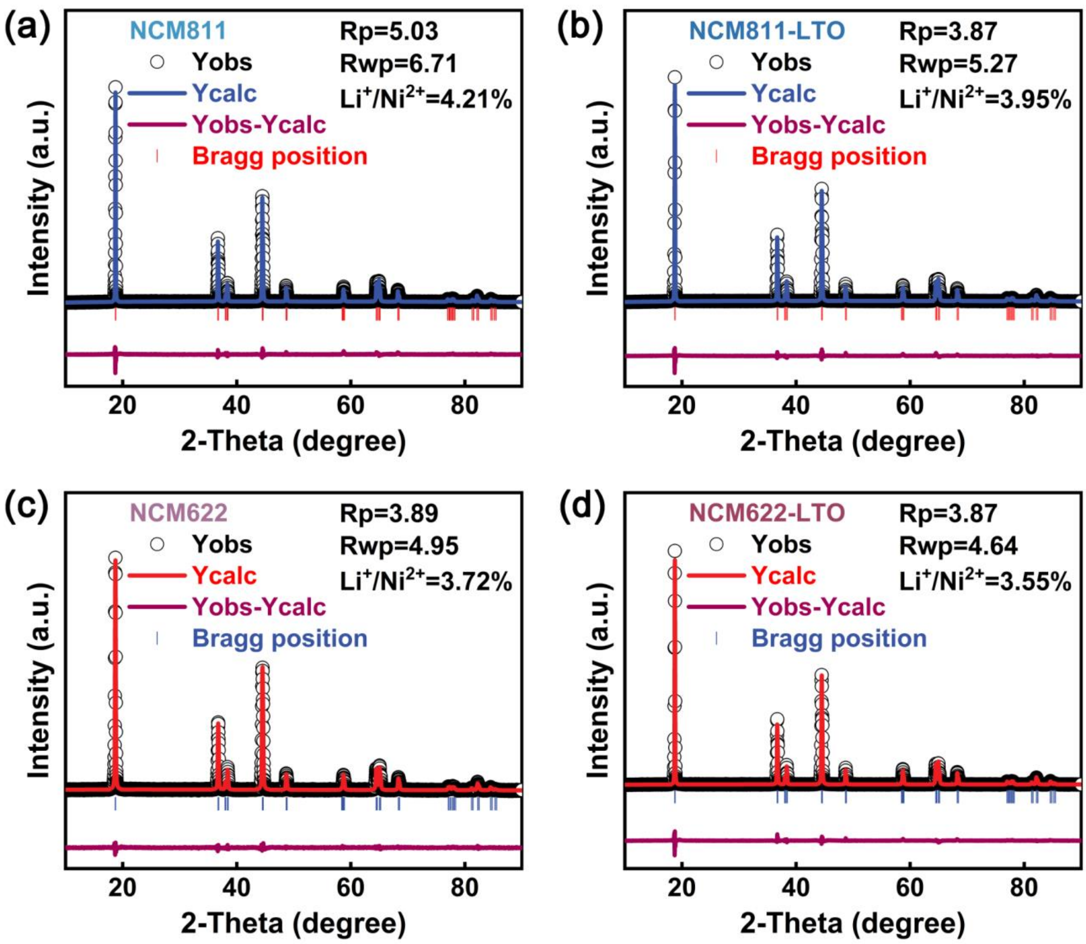

Figure S2. XRD refinement results for (a) NCM811, (b) NCM811-LTO, (c) NCM622 and (d) NCM622-LTO.

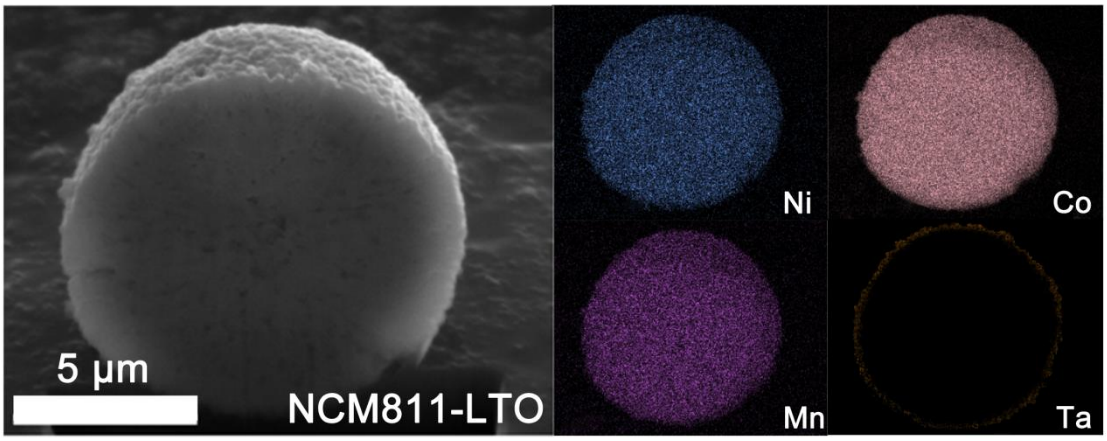

Figure S3. Cross-section SEM image combined with element mappings (Ni, Co, Mn, Ta) of the as-prepared NCM811-LTO.

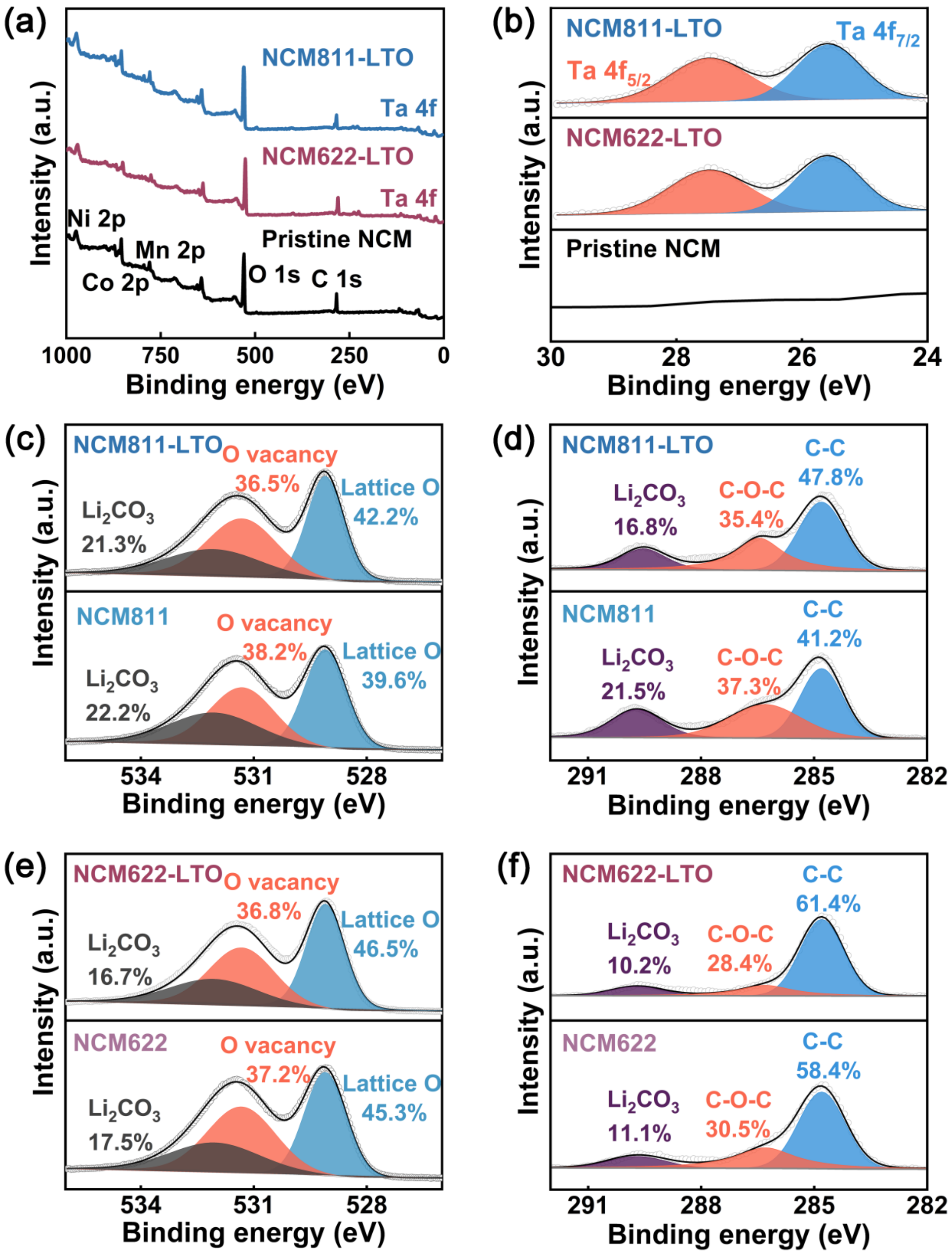

Figure S4. X-ray photoelectron spectra (XPS): (a)(b) full spectra and Ta 4f of NCM811-LTO, NCM622-LTO and pristine NCMs, respectively; (c)(d) O 1s and C 1s of the NCM811 and NCM811-LTO samples; (e)(f) O 1s and C 1s of the NCM622 and NCM622-LTO samples.

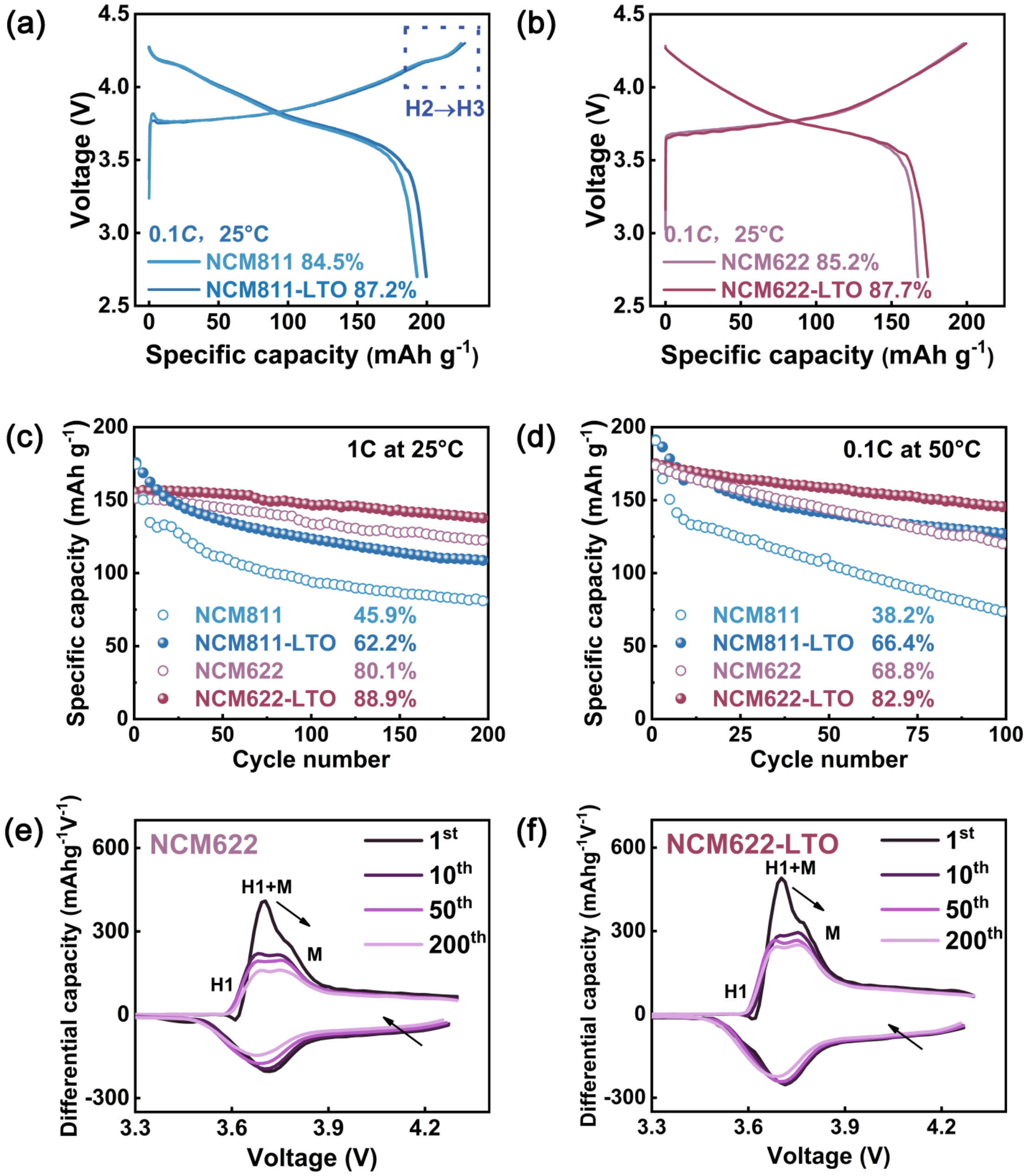

Figure S5. (a)(b) Initial charge/discharge curves for all the as-prepared cathodes including NCM811, NCM811-LTO, NCM622 and NCM622-LTO; (c)(d) cycling performances for the as-prepared cathodes at 1C (25 °C) and 0.1C (50 °C), respectively; (e)(f) dQ/dV curves in the range of 2.7–4.3 V for the NCM622 and NCM622-LTO cathodes, respectively.

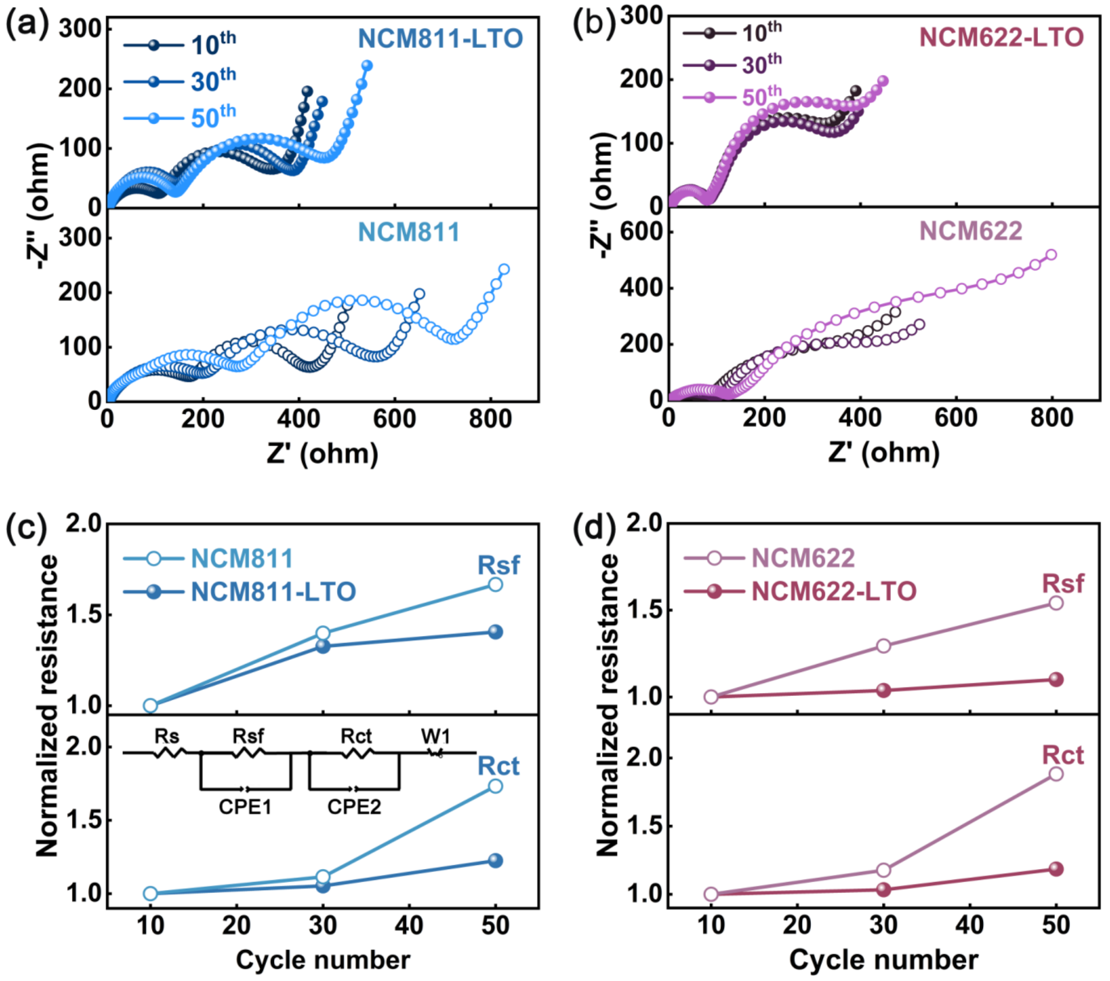

Figure S6. (a)(b) Nyquist plots of all the as-prepared cathodes including NCM811, NCM811-LTO, NCM622 and NCM622-LTO at different charged states with 10, 30 and 50 cycles, respectively; (c)(d) normalized resistance plots of R_{sf} and R_{ct} for the as-prepared samples including NCM811, NCM811-LTO, NCM622 and NCM622-LTO.

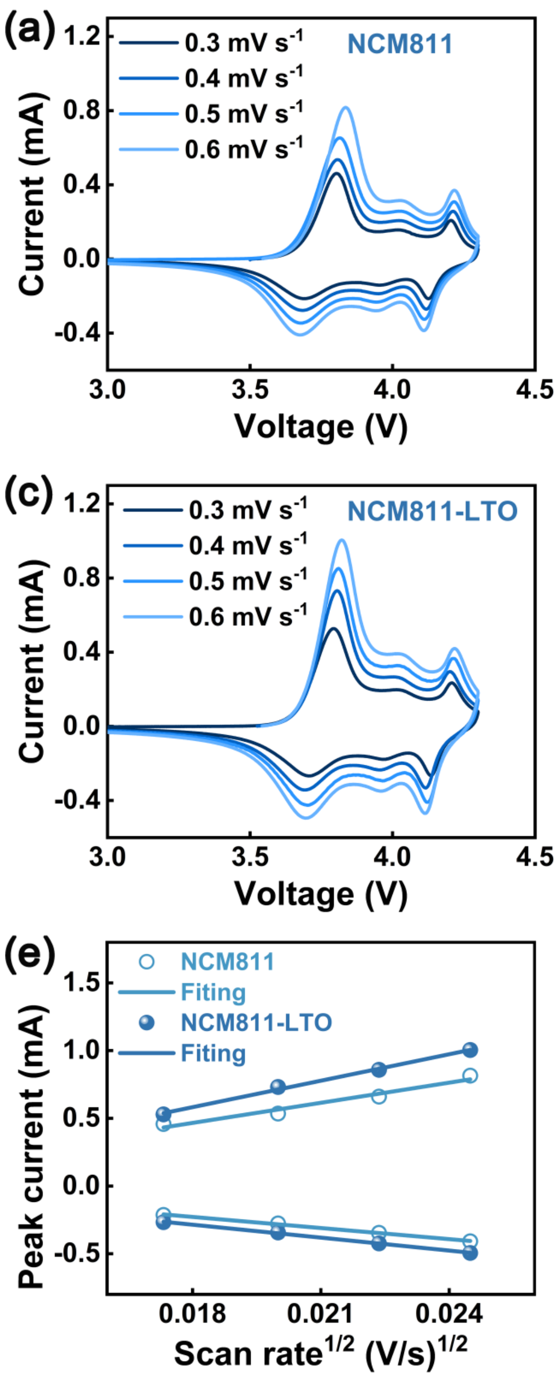

Figure S7. (a)–(d) Cyclic voltammetry (CV) curves with different scan rates for the as-prepared NCM811, NCM622, NCM811-LTO and NCM622-LTO cathodes, respectively; (e)(f) linear relationship fitting of I_p–$v^{1/2}$ for the NCM811, NCM811-LTO, NCM622-LTO and NCM622-LTO, respectively.

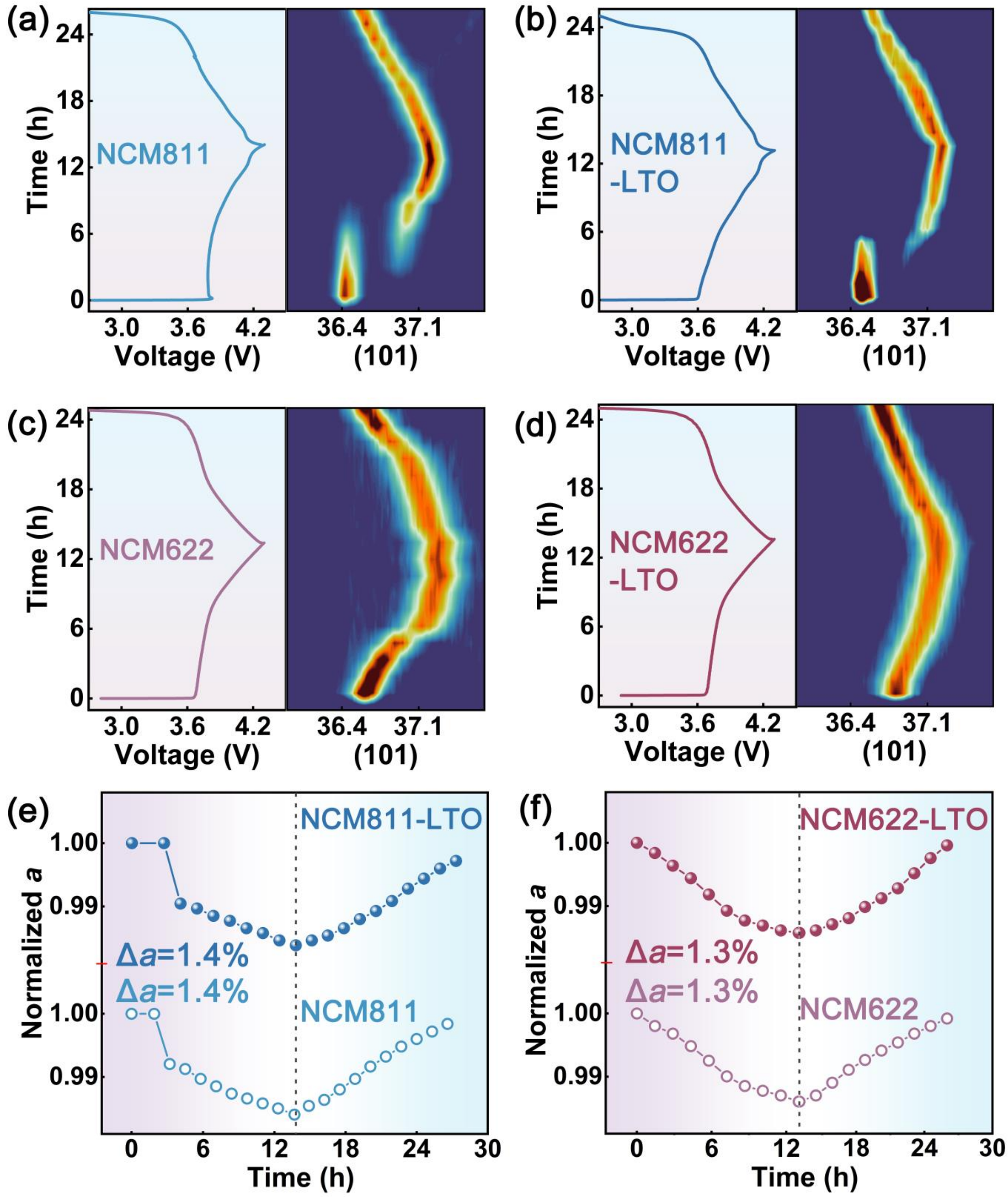

Figure S8. In situ XRD patterns of the (101) diffraction peaks in the initial cycle for (a) NCM811, (b) NCM811-LTO, (c) NCM622 and (d) NCM622-LTO; normalized evolutions of the lattice parameter *a*: (e) NCM811/NCM811-LTO, (f) NCM622/NCM622-LTO.

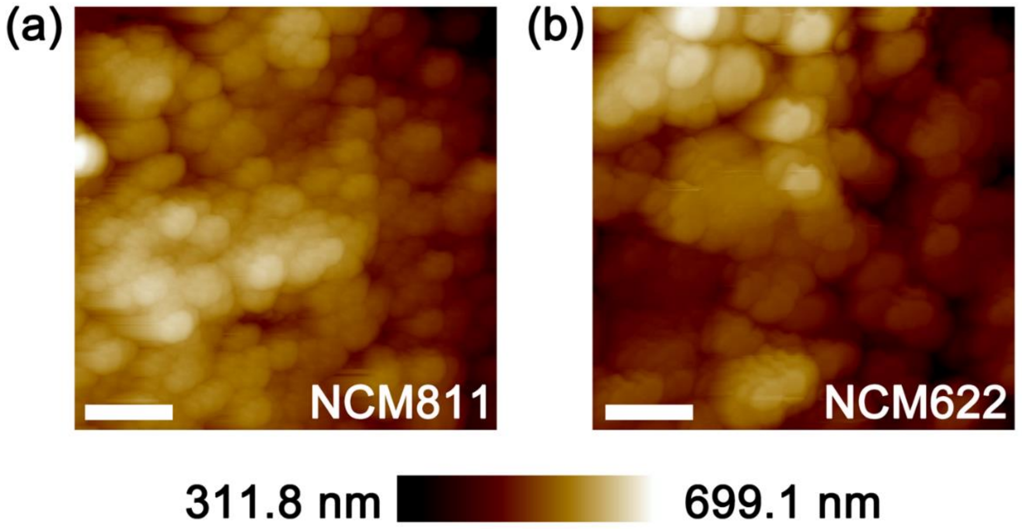

Figure S9. Morphological images with scale bar of 1 μm of (a) NCM811 and (b) NCM622 collected by AFM technique.

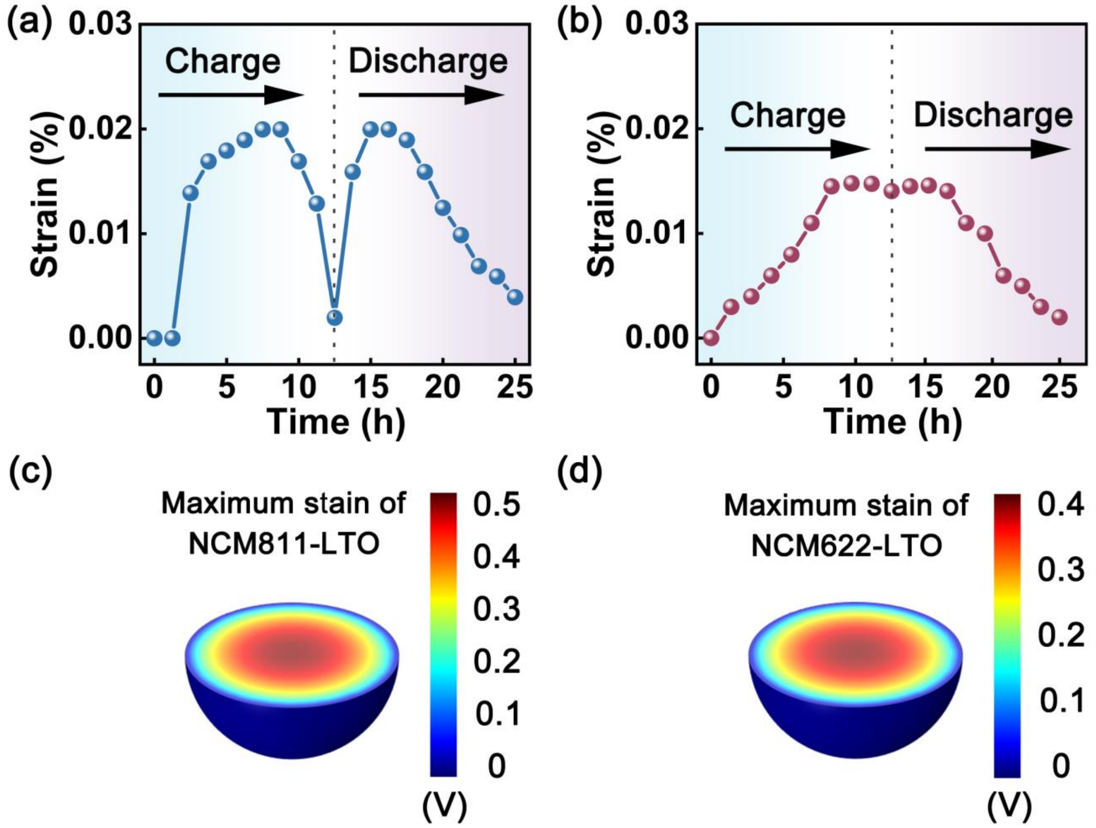

Figure S10. Strain curves of LTO-modified samples in the initial cycle: (a) NCM811-LTO, (b) NCM622-LTO; finite element simulation results of the piezoelectric potential distributed in pure LTO induced by Ni-rich samples at maximum strain state: (c) NCM811-LTO, (d) NCM622-LTO.

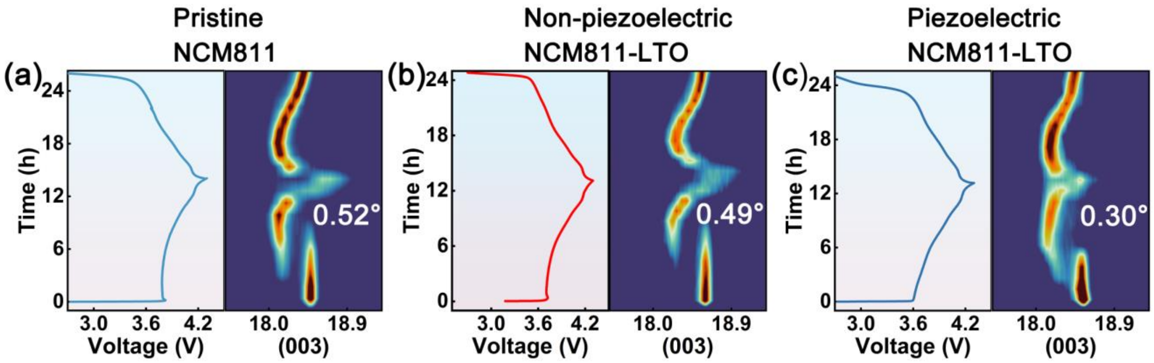

Figure S11. In situ XRD patterns of the (003) diffraction peaks in the initial cycle for (a) pristine NCM811, (b) non-piezoelectric NCM811-LTO and (c) piezoelectric NCM811-LTO.

Comparing the (003) diffraction peak shift among the three electrodes, it was clear that the stabilized structure of bulk Ni-rich cathode could be mainly attributed to the piezoelectric effect of LTO layer, which alleviated the lattice change caused by rapid Li^+ extraction through the induced piezoelectric field with inverse orientation. Though the influence of physical decoration on electrode stability has been extensively reported by previous researchers,[4,5] the pure LTO surface modification without piezoelectric characteristic contributed only slightly to the structure stabilization (0.49° *vs.* 0.52°).

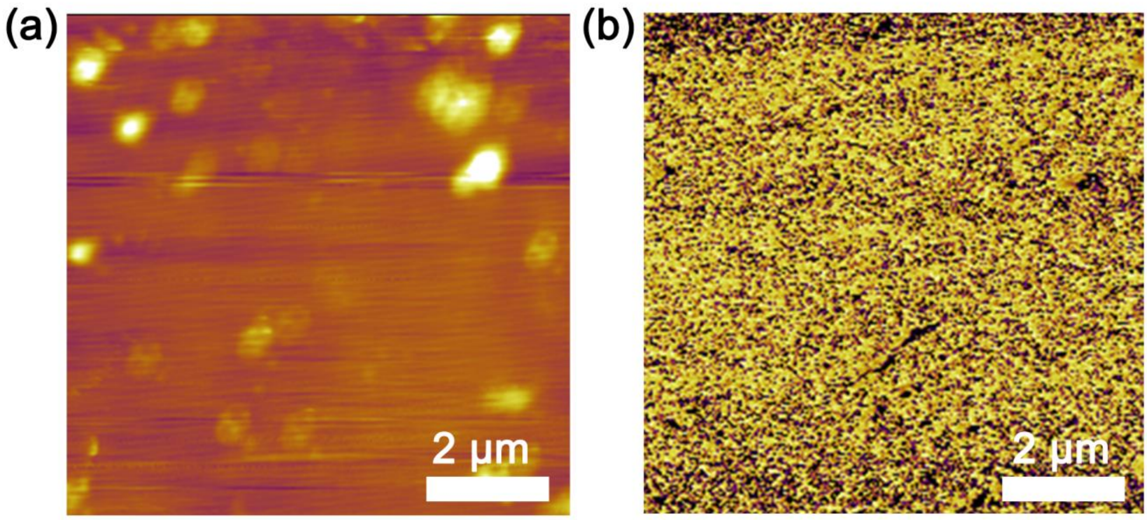

Figure S12. (a) Morphological and (b) phase images of the pure LTO powder tested by PFM with an applied voltage of 5 V.

As demonstrated in the PFM image, a low applied electric field (5 V) could not polarize LTO, implying that the influence of the external electric field (with voltage range of 2.7–4.3 V) on LTO upon cycling could be ignored.

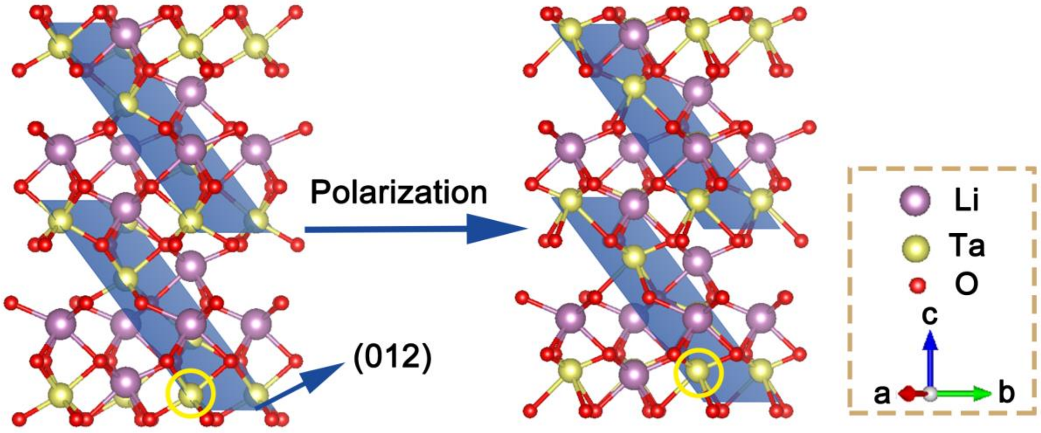

Figure S13. Schematic diagram for the lattice position variation of Ta atom consisted in (012) plane of LTO crystalline structure.

LTO is typically in paraelectric phase when the temperature exceeds its Curie temperature (665 °C), wherein Li^{+} ions locate in two equilibrium positions on both sides of the oxygen plane and Ta^{5+} ions distribute in the center of Ta-O octahedrons.[6] Nevertheless, Li^{+} ions leave the original equilibrium positions and Ta^{5+} ions deviate from the center of octahedrons under Curie temperature, which explains the spontaneous polarization of LTO (ferroelectric phase). As could be observed from the schematic diagram of LTO crystalline structure, the (012) lattice plane of LTO was only populated by Ta atoms, which was naturally utilized to reflect the polarization in LTO.

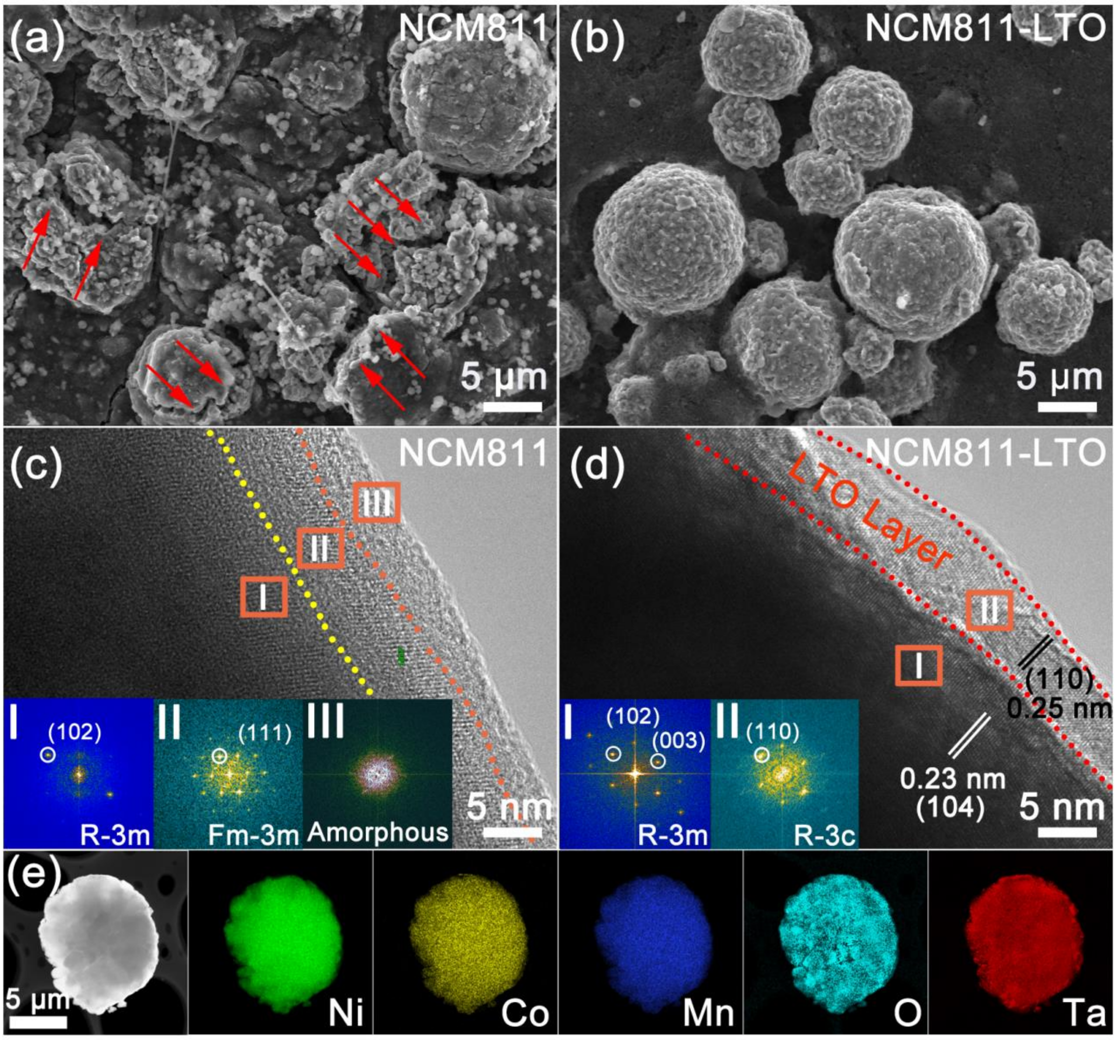

Figure S14. SEM images of (a) NCM811 and (b) NCM811-LTO after 200 cycles; HRTEM images of (c) NCM811 and (d) NCM811-LTO after 200 cycles; (e) element mapping images (Ni, Co, Mn, O, Ta) of the NCM811-LTO after 200 cycles.

Table S1. Calculated lattice parameters by Rietveld refinement for the as-synthesized electrodes.

Sample	$\boldsymbol{a = b}$ (Å)	$\boldsymbol{c}$ (Å)	$\boldsymbol{V}$ (Å^3)	$\boldsymbol{Z_{ox.}}$	$\boldsymbol{S_{TMO_6}}$ (Å)	$\boldsymbol{I_{LiO_6}}$ (Å)
**NCM811**	2.8695	14.1867	101.1600	0.2575	2.1528	2.5761
**NCM811-LTO**	2.8698	14.1893	101.2030	0.2579	2.1296	2.5902
**NCM622**	2.8678	14.2112	101.2153	0.2553	2.2178	2.5191
**NCM622-LTO**	2.8681	14.2158	101.2693	0.2554	2.2157	2.5228

Compared to the pristine NCM622, the c-axis of pristine NCM811 was reduced, which was caused by the cooperative replacement of Ni^{2+} (0.69 Å) and Mn^{4+} (0.53 Å) at 3b position in octahedron by Ni^{3+} (0.56 Å) ions.[7] Here, $Z_{ox.}$ was the atom coordination of oxygen atoms, S_{TMO_6} was the thickness of the TMO_6 slab and calculated by the $2(1/3 - Z_{ox.})c$, meanwhile the thickness (I_{LiO_6}) of LiO_6 slab was calculated by the $I_{LiO_6} = c/3 - S_{TMO_6}$. Because LiO_6 slab provides the Li^+ diffusion and its thickness plays important role for the Li^+ diffusion during electrochemical cycling, it was suggested the Li^+ diffusion kinetics in NCM811 was better than NCM622 since the former has a larger I_{LiO_6} than the latter. In addition, the values of the I_{LiO_6} of pristine Ni-rich cathodes have been increased with LTO coating, indicating the modification effect of LTO coating on the Li^+ diffusion ability of Ni-rich cathodes.

Table S2. Electrochemical performances for the as-synthesized electrodes.

Performance	NCM811	NCM811-LTO	NCM622	NCM622-LTO
Initial discharge capacity (mAh g^{-1})	193.0	199.6	167.4	174.1
Initial coulombic Efficiency (%)	84.5	87.2	85.2	87.7
Capacity retention (%) (0.1C, 25 °C, 200 cycles)	59.7	71.8	79.4	88.8
Capacity retention (%) (1C, 25 °C, 200 cycles)	45.9	62.2	80.1	88.9
Capacity retention (%) (0.1C, 50 °C, 200 cycles)	38.2	66.4	68.8	82.9
Voltage fading (mV n^{-1}) (0.1C, 25 °C, 200 cycles)	0.53	0.45	0.16	0.09

Table S3. EIS fitting results for the as-synthesized electrodes.

Sample		10th	30th	50th
**NCM811**	R_{sf} (Ω)	168	235	280
	R_{ct} (Ω)	254	283	440
**NCM811-LTO**	R_{sf} (Ω)	101	134	142
	R_{ct} (Ω)	245	258	300
**NCM622**	R_{sf} (Ω)	85	110	131
	R_{ct} (Ω)	255	300	480
**NCM622-LTO**	R_{sf} (Ω)	80	83	88
	R_{ct} (Ω)	243	168	235

Table S4. Lithium diffusion coefficient ($cm^2\ s^{-1}$) for the as-synthesized electrodes.

Process	NCM811	NCM811-LTO	NCM622	NCM622-LTO
**Charge**	5.14×10^{-11}	8.09×10^{-11}	1.17×10^{-11}	2.07×10^{-11}
**Discharge**	1.56×10^{-11}	2.12×10^{-11}	0.43×10^{-11}	0.93×10^{-11}

Formula S1:

$$\varepsilon = \frac{\Delta d}{d} \qquad (2)$$

here ε was the strain produced by the interplanar spacing, Δd was the change in interplanar spacing, and d is the interplanar spacing.

Formula S2:

$$E = \frac{\sigma}{\varepsilon} \qquad (3)$$

here E is the Young's modulus of the samples, σ is the stress, and ε is the strain.

References in SI:

[1] G. Kresse, D. Joubert, *Phy. Rev. B* **1999**, 59, 1758.

[2] G. Henkelman, B. P. Uberuaga, H. JoNsson, *J. Chem. Phy.* **2000**, 113, 9901.

[3] Y. Gao, Q. Liu, B. Xu, *ACS Nano* **2016**, 10, 5431.

[4] C. Xu, K. Marker, J. Lee, A. Mahadevegowda, P. J. Reeves, S. J. Day, M. F. Groh, S. P. Emge, C. Ducati, B. Layla Mehdi, C. C. Tang, C. P. Grey, *Nat. Mater.* **2021**, *20*, 84.

[5] S. Yin, W. Deng, J. Chen, X. Gao, G. Zou, H. Hou, X. Ji, *Nano Energy* **2021**, *83*, 105854.

[6] A. M. Glass, M. E. Lines, *Phys. Rev. B* **1976**, *13*, 180.

[7] W. Lee, S. Muhammad, T. Kim, H. Kim, E. Lee, M. Jeong, S. Son, J. H. Ryou, W. S. Yoon, *Adv. Energy Mater.* **2018**, 8, 1701788.
